# Pain care for patients with epidermolysis bullosa: best care practice guidelines

**DOI:** 10.1186/s12916-014-0178-2

**Published:** 2014-10-09

**Authors:** Kenneth R Goldschneider, Julie Good, Emily Harrop, Christina Liossi, Anne Lynch-Jordan, Anna E Martinez, Lynne G Maxwell, Danette Stanko-Lopp

**Affiliations:** Pain Management Center, Department of Anesthesiology, Cincinnati Children's Hospital Medical Center, Cincinnati, Ohio USA; Lucille Packard Children's Hospital, Department of Anesthesia (by courtesy, Pediatrics), Stanford University, Stanford, California USA; Helen and Douglas Hospices, Oxford and John Radcliffe Hospital, Oxford, USA; University of Southampton, Southampton, UK; Great Ormond Street Hospital for Children NHS Trust, London, UK; Pain Management Center and Division of Behavioral Medicine and Clinical Psychology, Cincinnati Children's Hospital Medical Center, Cincinnati, Ohio USA; National Paediatric Epidermolysis Bullosa Centre, Great Ormond Street Hospital NHS Foundation Trust, London, UK; Department of Anesthesiology and Critical Care, Children's Hospital of Philadelphia, Philadelphia, Pennsylvania USA; James M. Anderson Center for Health Systems Excellence, Cincinnati Children's Hospital Medical Center, Cincinnati, Ohio USA

**Keywords:** Epidermolysis bullosa, Pain, Practice guidelines, RDEB, DEBRA, Acute pain, Chronic pain, Recessive dystrophic epidermolysis bullosa, Dystrophic Epidermolysis Bullosa Research Association International

## Abstract

**Background:**

Inherited epidermolysis bullosa (EB) comprises a group of rare disorders that have multi-system effects and patients present with a number of both acute and chronic pain care needs. Effects on quality of life are substantial. Pain and itching are burdensome daily problems. Experience with, and knowledge of, the best pain and itch care for these patients is minimal. Evidence-based best care practice guidelines are needed to establish a base of knowledge and practice for practitioners of many disciplines to improve the quality of life for both adult and pediatric patients with EB.

**Methods:**

The process was begun at the request of Dystrophic Epidermolysis Bullosa Research Association International (DEBRA International), an organization dedicated to improvement of care, research and dissemination of knowledge for EB patients worldwide. An international panel of experts in pain and palliative care who have extensive experience caring for patients with EB was assembled. Literature was reviewed and systematically evaluated. For areas of care without direct evidence, clinically relevant literature was assessed, and rounds of consensus building were conducted. The process involved a face-to-face consensus meeting that involved a family representative and methodologist, as well as the panel of clinical experts. During development, EB family input was obtained and the document was reviewed by a wide variety of experts representing several disciplines related to the care of patients with EB.

**Results:**

The first evidence-based care guidelines for the care of pain in EB were produced. The guidelines are clinically relevant for care of patients of all subtypes and ages, and apply to practitioners of all disciplines involved in the care of patients with EB. When the evidence suggests that the diagnosis or treatment of painful conditions differs between adults and children, it will be so noted.

**Conclusions:**

Evidence-based care guidelines are a means of standardizing optimal care for EB patients, whose disease is often times horrific in its effects on quality of life, and whose care is resource-intensive and difficult. The guideline development process also highlighted areas for research in order to improve further the evidence base for future care.

**Electronic supplementary material:**

The online version of this article (doi:10.1186/s12916-014-0178-2) contains supplementary material, which is available to authorized users.

## Background

Inherited epidermolysis bullosa (EB) comprises a group of rare disorders, generally thought of as skin diseases. However, EB has multi-system effects and patients present with a number of both acute and chronic pain care needs [1]. Effects on quality of life are substantial [2],[3]. Due to its low prevalence, expertise in pain care for patients with this disease is often restricted to a few specialized care centers. Even then, evidence-based pain care is limited by a near absence of scientific literature specific to EB. This set of guidelines was requested by Dystrophic Epidermolysis Bullosa Research Association International (DEBRA International) to help standardize the approach to pain care for both adult and pediatric patients with EB in all parts of the world. Consequently, a group of clinical pain care experts from a few countries have come together to lend their experience to the limited scientific literature to create these guidelines.

The present guidelines on pain care for patients with EB are based on a review and synthesis of the available literature, guided by expert consensus and thoughtful application of theory. The guidelines are divided into four topics: psychological treatment of pain and pruritus, acute pain, chronic and recurrent pain, and special topics. The Psychological and Integrative Treatments section leads off because the information it contains applies to all the topics that follow. The Acute Pain section focuses on postoperative pain. The Chronic and Recurrent Pain section includes dressing changes, baths, skin pain and joint and other body pains. The Special Topics section includes pain care in infants with EB and pain care at the end of life. While not pain *per se*, itching is a major source of discomfort [4] and is also discussed.

The aim of the guidelines is to provide the user with information on pain care for children and adults with EB. These guidelines can be applied to all patients diagnosed with hereditary forms of EB. Patients with acquired forms are not included in the guidelines. The guidelines contain two types of guidance. Recommendations are graded and highlighted in text boxes at the end of the manuscript. Good Practice Points are located at the end of each topical section and summarize concepts and best practices, based on the clinical experience of the guidelines development group.

The daily routine of many patients with severe forms of EB will have a number of painful events, each of which may suggest the need for intervention. However, addressing each one pharmacologically may lead to a level of sedation that prevents a meaningful level of productivity. It is important to discuss the needs of the patient and the options for caring for those needs with the patient and family. Together, the patient, family and practitioner can transform the guidelines into an individualized care plan. It is to be expected that each patient’s needs will be dynamic; periodic review of the needs and goals will optimize care at each step of the patient's life.

Of note, the term `EB’ will be used throughout the text. However, four major types of EB are known and are categorized as Simplex (EBS), Junctional (JEB), dystrophic types (DEB) and Kindler Syndrome. A recent consensus statement was released that reclassified the subtypes further based on anatomic location within the skin and pattern of involvement, and discouraged the use of eponyms (with the exception of Kindler Syndrome). EBS is sub-typed as EBS suprabasal and EBS basal, JEB as JEB generalized and JEB localized, DEB as Recessive DEB and Dominant DEB (RDEB and DDEB, respectively) [5]. Additionally, there are at least 18 genes associated with the different types of EB [5]. The proposed new classification does not address the relation to painful conditions, and, thus, the four major types will be referenced throughout the guidelines. There is a wide range of severity within the types. EBS is usually due to autosomal dominant mutations in keratin 5 or 14 or in plectin and is often of milder severity. JEB can result from mutations in any of six different basement membrane components, is inherited as an autosomal recessive disorder, and can range from mild to fatal early in life. DEB can be in a mild dominant form (DDEB) or a more severe recessive one (RDEB), both due to mutations in collagen 7. The recommendations that follow are intended to be generally applicable to all patients with EB who experience pain. The pain conditions vary in prevalence among the types of EB, and readers will find relevant guidelines to apply in the care of the particular pain problems they and their patients face. Resources (for example, particular medications and formulations, trained medical, nursing and therapy personnel) will vary by location, and practitioners and families will, therefore, need to adapt the recommendations based on what is available in their locale. DEBRA International [6] is an optimal facilitating organization that can aid implementation of the guidelines by way of providing information, support and means of contacting expert care providers.

## Methods

In 2011, a multidisciplinary working group formed to develop best care practice guidelines for pain associated with EB. The group comprised representatives from nursing, medicine and psychology who were expert in the clinical care of patients with EB. Guidelines topics and subtopics were chosen by the multidisciplinary group with input from outside clinicians, based on issues presented in the literature, seen clinically and raised for discussion at DEBRA International Congress meetings. Individuals or small groups were assigned to the various subtopics. After relevant literature was reviewed, preliminary recommendations for each subsection were made. Recommendations were circulated by email among the entire panel of experts for review and input/feedback was incorporated. Initial citations were added or removed as required for accuracy and appropriateness, and criticism made of areas of weakness. Thus, the group formed the first iteration of expert consensus. The revised recommendations were recirculated to the group for review, to establish consensus more firmly. A panel of outside reviewers, comprising both clinicians and patients/families, then reviewed the document for comprehensibility, omissions and applicability.

Following external review (see Acknowledgements for reviewers), funding became available and a subsequent systematic evidence review was conducted by a trained methodologist (author DSL). Clinical questions and the systematic search strategy were developed, based on the guideline topics and subtopics from the working group. The population of interest comprises patients with pain who have any variant of EB. The interventions included, but were not limited to, pharmacological, holistic, psychological, psychical therapy and/or environmental interventions. Outcomes of interest were improvement in symptom control (for example, pain, itch) and level of function.

Systematic literature searches were conducted using MedLine, CINAHL, PsycInfo and *The Cochrane Library*. Search terms for `epidermolysis bullosa’ and `pain’, `regional anesthesia’, or `nerve block’ were used as medical subject headings and/or keywords in all databases. The search was only limited to articles published in English. No other restrictions, limits or filters were used. Publication dates were not restricted for any topic or subtopic, as the diagnosis is rare and there were few studies of higher level, directly-related evidence (that is, systematic reviews, meta-analyses, randomized controlled trials). Reference lists were searched to identify studies. Studies submitted by the working group and used in the initial consensus processes were also reviewed. The most recent systematic search for pediatric acute, chronic, or recurrent pain management in EB patients was conducted in December 2011.

**Figure 1:**
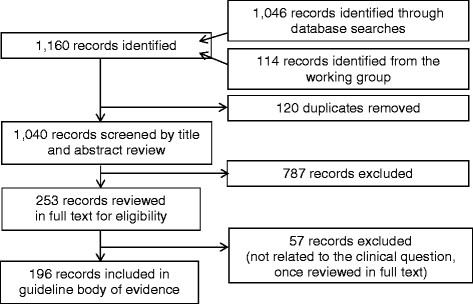
**Flow of information through the evidence evaluation process.**

Citations from the evidence searches were reviewed by title and abstract for potential inclusion, regardless of study design (n = 1,061). Evidence related to the clinical questions from systematic reviews, meta-analyses and RCTs as well as observational studies, case reports and expert opinion articles were reviewed (see Figure 1). A total of 57 references were found and evaluated that were specific to the cross match of EB and the pain-related keywords (see above), of which only 8 were included into specific recommendations. The rationale for including and excluding these references is found in Table 1 and in Additional file 1.

**Table 1:** **EB-specific articles used in producing recommendations**

Study citation	Study type	Population (setting, patients)	Intervention/Comparison groups	Outcomes
Chiu 1999 [68]	Case report	Country: Canada Setting: Children’s Hospital Age: 11 years Gender: Male Patient with Severe JEB	Amitryptiline (25 mg at night) was prescribed and patient started on a program of cognitive behavioral training (hypnotic imagery, distraction). Oral midazolam (7.5 mg) was initiated 20 minutes prior to bath or dressing change	Pain management
Goldschneider 2010 [41],[42]	Review articles	Country: United States Setting: Children’s Hospital Patients with EB	Pain management and prevention	Pain management
Herod 2002 [44]	Review article	Country: England (London) Setting: Children’s Hospital Patients with EB	General pain management	Pain management
Mellerio 2007 [152]	Review article	Country: United States, England, Chile Setting: Hospital Patients with EB	Medical management	General pain management
Saroyan 2009 [156]	Case report	Country: United States Setting: Hospital Female infant with EBS, severe, Dowling-Meara subtype	Use of IV ketamine given orally Oral administration of IV ketamine (10 mg/mL, Monarch Pharmaceuticals) at a starting dose of 0.5 mg (0.125 mg/kg/ dose) Over four days, the dose was titrated to 3 mg (0.75 mg/kg/dose) in response to observed effect	Achieve analgesia during painful dressing changes
van Scheppingen 2008 [4]	Qualitative study (Interviews)	Country: Netherlands Setting: Center for Blistering Diseases Age: 6 to 18 years Children with different (sub)types of EB)	Interviews conducted at homes or in hospital Questions explored were: (i) What problems do children with EB actually experience as being the most difficult? (ii) What is the impact of these problems on their daily life? (iii) Are there differences in experiences between mildly and severely affected children?	Themes of pain for severe disease (generalized blistering with motion impairment) and for mild disease (localized blistering or generalized blistering without motion impairment).
Watterson 2004 [74]	Case report	Country: United States Setting: Hospital Children with EB using peripheral opioids	Topical morphine gel applied to the most painful areas of skin at that time for each child	Pain scores

EB, epidermolysis bullosa, EBS, epidermolysis bullosa simples; JEB, junctional epidermolysis bullosa.

All included articles were critically appraised using evidence appraisal forms from the LEGEND (Let Evidence Guide Every New Decision) evidence evaluation system [7],[8]. The methodologist assessed risk of bias in the included studies by evaluating quality for all individual studies by domain and study design. The quality levels for included studies were recorded (see Table 2). The reader will find the evidence level for each article used to directly support each recommendation noted after its citation in the Reference section of the Summary of recommendations (Table 3). Age specificity of each recommendation is also noted. Data were collected on descriptive characteristics of patients, characteristics of the pain management interventions, and associated outcomes of reported pain management interventions. Using the Grade for the Body of Evidence tool in the LEGEND system [7] provided objective criteria for evaluating evidence related to each recommendation (see Table 4).

**Table 2:** **Evidence levels**[8]

Quality level	Definition
1a^a^ or 1b^a^	Systematic review, meta-analysis, or meta-synthesis of multiple studies
2a or 2b	Best study design for domain
3a or 3b	Fair study design for domain
4a or 4b	Weak study design for domain
5a or 5b	General review, expert opinion, case report, consensus report, or guideline
5	Local Consensus

^a^a = good quality study; b = lesser quality study.

**Table 3:** **Summary of recommendations**

	Application	Level of recommendation	Target age group	Key references (evidence grade)
**A. Psychological therapies offer effective approaches to management of chronic and acute pain as well as itching.**				
	For chronic pain management use cognitive behavioral therapy (CBT).	B	All	Gerik 2005 [13] (5a), Palermo 2005 [17] (5a)
	For acute pain management, offer the patient distraction, hypnosis, visualization, relaxation or other forms of CBT	B	All	Green 2005 [14] (5a), Uman 2006 [21]
	Consider habit reversal training, and other psychological techniques for management of pruritus	C	All	Chida 2007 [29] (1a), Ehlers 1995 [32] (2b), Azrin 1973 [30] (4b), Hagermark 1995 [28] (5a), Rosenbaum 1981 [31] (5a)
**B. Postoperative pain can be handled as for other patients in the same setting, with modifications.**				
	Basic perioperative assessment and pain treatments should be used as for non-EB patients, with modification	A	All	Goldschneider 2010 [41] (5a), Goldschneider 2010b [42] (5a)
	Transmucosal (including intranasal fentanyl and transbuccal opioids) should be considered for short procedures and pain of brief duration when intravenous and enteral routes are unavailable	B	All	Manjushree *et al*., 2002 [45] (2b); Borland *et al*., 2007 [46] (2b); Desjardins *et al*., 2000 [47] (2a)
	Perioperative opioid use must account for preoperative exposure, with appropriate dose increases to account for tolerance	B	All	Hartrick 2008 [56] (1a), Mhuircheartaigh 2009 [55] (1a), Viscusi 2005 [54] (2a)
	Regional anesthesia is appropriate for pain resulting from a number of major surgeries. Dressing of catheters must be non-adhesive and monitored carefully	C	All	Diwan 2001 [51] (5a), Doi 2006 [53] (5b), Englbrecht 2010 [52] (5a), Kelly 1988 [48] (5b), Sopchak 1993 [49] (5a), Yee 1989 [50] (5a)
**C. Skin wounds and related pain are the hallmark of EB of most subtypes. Prevention and rapid healing of wounds through activity pacing, optimal nutrition and infection control are important. A number of pharmacologic treatments are available**				
	Maintain optimal nutrition and mobility and treat infections as indicated	D	All	Denyer 2010 [57] (5a)
	Consider topical therapies for pain	C	All	Cepeda 2010 [77] (1a), Lander 2006 [76] (1a), LeBon 2009 [73] (1a), Twillman 1999 [72] (5a), Watterson 2004 [74](5a)
	Systemic pharmacologic therapy should be adapted to treat both acute and chronic forms of skin pain	B	All	Noble 2010 [59] (1a), Moore, 2011 [ 67] (1a), Nicholson 2009 [65] (1a)
	Monitor potential long-term complications of chronically administered medications	C	Pediatric	Huh 2010 [62] (4a), Camilleri 2011 [66] (5a), Chiu 1999 [68](5a), Cruciani 2008 [63] (5a), Gray 2008 [69] (5a)
**D. Baths and dressing changes require attention to both pain and anxiety**				
	Anxiolytics and analgesics should be used for procedural pain and fear. Care must be taken when combining such medications due to cumulative sedative effects	B	All	Bell 2009 [85] (1a), Blonk 2010 [84] (1b), Ezike 2011 [82] (2a), Desjardins 2000 [47] (2a), Borland 2007 [46] (2b), Manjushree 2002 [45] (2b), Humphries 1997 [83] (2b), Wolfe 2010 [81] (5a), Ugur 2009 [86] (5a)
	Cognitive behavioral techniques should be implemented as the child becomes old enough to use them effectively. Specifically, distraction should be used for younger children	B	All	Green 2005 [14] (5a); Gerik 2005 [13] (5a), Palermo 2005 [17] (5a)
	Environmental measures such as adding salt to the water to make it isotonic and keeping the room warm are recommended	B	All	Arbuckle 2010 [78] (5a), Cerio 2010 [79] (5a), Peterson (Poster) 2011 [80] (5b)
**E. EB affects the gastrointestinal tract in its entirety. Pain from ulcerative lesions responds to topical therapy. GERD and esophageal strictures have nutritional as well as comfort implications and should be addressed promptly when found. Maintaining good bowel habits and reducing iatrogenic causes of constipation are crucial.**				
	Topical treatments are recommended for oral and perianal pain	C	All	Ergun 1992 [98] (4a), Travis 1992 [97] (4b), Marini 2001 [99] (5a), Buchsel 2008 [100] (5a), Buchsel 2003 [101] (5b), Cingi 2010 [102] (2a)
	Therapy should be directed to manage gastroesophageal reflux and esophageal strictures using standard treatments	C	All	Freeman 2008 [95] (4a)
	Constipation should be addressed nutritionally, with hydration and addition of fiber in the diet to keep stool soft, by minimizing medication-induced dysmotility and with stool softeners	C	All	Belsey 2010 [112] (1a), Freeman 2008 [98] (4a), Hanson 2006 [113] (4a)
**F. Bone pain treatment must account for factors that include nutrition, mobility, potential occult fractures and is treated by combinations of nutritional, physical, pharmacologic and psychological interventions.**				
	Joint pain should be treated with mechanical interventions, physical therapy, CBT and surgical correction	C	All	Bruckner 2011 [126] (4b), Gandrud 2003 [131] (4b), Martinez 2010 [125] (5a), Lacativa 2010 [127] (5a), Tilg 2008 [128] (5a), Noguera 2003 [130] (5a), Falcini 1996 [132] (5a)
	Osteoporosis should be treated to reduce pain in EB	D	All	Levis 2012 [129] (1a), Martinez 2010 [125] (5a)
	Back pain should be addressed with standard multi-disciplinary care	C	All	Chou, *et al*., 2007 [133] (5a)
**G. Corneal abrasions are common in EB, prevention and supportive care are appropriate**				
	Care should include general supportive and analgesic care, protecting the eye from further damage, and topical therapies	C	All	Watson, 2012 [136] (1a), Calder 2005 [138] (1a)
**H. Pain in infants is as widespread as in any other age, but unique pharmacologic, developmental and physiologic issues must be accounted for in infants with all types of EB**				
	Assess patients as needed and prior to and after interventions; health care workers should use validated measures. (Grade: A)	A	Infants	Gibbins 2014 [139] (2a) Stevens 2014 [140] (2a), Hummel 2008 [141] (2a), Krechel 1995 [142] (2b), Lawrence 1993 [143] (2b), Manworren 2002 [144] (2a)
	Sucrose solutions should be administered for mild to moderate pain alone or as an adjunct	B	Young infants	Harrison 2010 [150] (1a), Yamada 2008 [149] (1a) Cignacco 2012 [151] (2a)
	Standard analgesics should be used in infants as in older patients with careful attention to dosing and monitoring	B	Infants	Tremlett 2010 [153] (5a), MacDonald 2010 [154] (5b)
**I. End of Life pain care is an expected part of care for EB, which in many cases is life-limiting in nature. All basic principles of palliative care apply as they do for other terminal disease states.**				
	Assess and manage physical, emotional and spiritual suffering of the patient, while providing support for the whole family	A	All	Craig 2007 [165] (5a), AAP 2000 [166] (5b), WHO [167] (5b)
	Opioids are the cornerstone of good analgesia in this setting. Opioid rotation may need to be considered to improve analgesia and reduce side effects, and adjuncts may need to be added	B	All	Eisenberg 2009 [174] (1a), Quigley 2010 [172] (1a), Davies 2008 [61] (4a), Bruera 1996 [171] (4b), Watterson 2005 (5b)
	Consider targeted medication for neuropathic pain when pain proves refractory to conventional therapies	D	All	Allegaert 2010 (5a), Saroyan 2009 (5a). Clements 1982 (5a), Watterson 2005 [87] (5b)
	Continuous subcutaneous infusion of combinations of medication is an option when parenteral therapy is needed in the terminal phase	C	All	Reymond 2003 [178] (2b), O’Neil 2001 [176] (5a), Watterson 2005 [87] (5b)
	Where needed, breakthrough medication can be given transmucosally (oral or nasal) for rapid onset and avoidance of the enteral route	B	All	Zeppetella 2009 [182] (1a)
**J. A combination of environmental, cognitive-behavioral and pharmacologic therapies are available for use for EB-related pruritus, which can be a severe symptom of the disease.**				
	Use environmental and behavioral interventions for itch control	C	All	Nischler 2008 [183] (5b), EB Nurse Website [187] (5b)
	Antihistamines are recommended and can be chosen depending upon desirability of sedating effects	D	All	Ahuja 2011 [188] (2b), Goutos 2010 [186] (3a)
	Gabapentin, pregabalin, TCA, SNRIs and other non-traditional antipruritics agents should be strongly considered for itch treatment	C	All	Goutos 2010 [186] (3a), Ahuja 2013 [189] (2a), Murphy 2003 [197] (2a)

EB, epidermolysis bullosa; GERD, gastroesophageal reflux disease; SNRI, serotonin norepinephrine reuptake inhibitors; TCA, tricyclic antidepressant.

**Table 4:** **Table for judging the strength of a recommendation**[8]

Dimension	Definition
Grade of the body of evidence	High, Moderate, Low, Not Assignable
Safety/harm **(side effects and risks)**	Minimal, Moderate, Serious
Health benefit to patient	Significant, Moderate, Minimal
Burden on patient to adhere to recommendation	Low, Unable to determine, High
Cost-effectiveness to healthcare system	Cost-effective, Inconclusive, Not cost-effective
Directness of the evidence for this target population	Directly relates, Some concern of directness, Indirectly relates
Impact on morbidity/mortality or quality of life	High, Medium, Low

Given the paucity of data for statistical analysis, it was deemed appropriate to conduct a face-to-face consensus meeting on 4, 5 May 2012 in Cincinnati, OH. The LEGEND tool for Judging the Strength of a Recommendation (see Table 4) was used as a guide for finalizing recommendation statements by discussing the body of evidence and discussing safety/harm and other dimensions [7]. The overall GRADE (A, B, C, D) for each recommendation statement was then determined, based on this process and the established criteria for GRADE [9],[10].

### Updating procedure

The guidelines will be updated every three years after the first version. If new relevant evidence is detected before the update, the information will be published on the web page for DEBRA International that is dedicated to clinical the Pain guidelines [11].

## Results and discussion

### Psychological and integrative approaches

#### Introduction

A biopsychosocial approach emphasizing medical, psychological and physical therapies for pain management has been suggested to be the most useful for adults and children with acute and chronic pain [12]. This approach has also been advocated for EB patients [3] and should be initiated from early in youth and modified with maturation. It is recommended that psychological interventions be used in conjunction with physical and pharmacologic therapies.

#### Types of therapies

Psychological therapies for pain management have been shown to modify pain intensity, reduce related distress, decrease pain-related functional disability and improve pain coping. Painful experiences amenable to treatment include acute pain related to procedures (for example, whirlpool treatments) or medical routines (for example, bandage changes, bathing), and chronic pain conditions such as headache, abdominal pain or other disease-related conditions. Psychological therapies include cognitive behavioral therapy (CBT), hypnosis, biofeedback and relaxation training, among others. CBT focuses on changing the catastrophic thinking and negative emotions surrounding pain as well as modifying lifestyle to promote wellness behavior despite chronic pain [13]. Hypnosis is a psychological state of heightened awareness and focused attention, in which critical faculties are reduced and susceptibility and receptiveness to ideas are greatly enhanced [14]. Relaxation training usually includes techniques, such as diaphragmatic breathing, muscle relaxation and visualization/imagery, that assist in adaptive coping via distraction, reduced emotional arousal and activation of the parasympathetic nervous system. While these interventions are frequently patient-focused, CBT can also include parent/family training related to behavioral management, reducing caregiver distress and enhancing environmental factors necessary for positive coping [15].

As an adjunct to relaxation training, biofeedback monitors physiological functions (for example, heart rate, muscle tension, temperature) in order to improve control of bodily processes that might ease chronic pain. Frequently, sensors are attached to the skin by adhesive, which is relatively contraindicated in EB. Clip on pulse oximeter probes can be safely used in patients with EB to track heart rate, and provide an alternative method of biofeedback.

#### Efficacy of psychological interventions in chronic and acute pain management

Psychological therapies have been shown to be effective in decreasing pain intensity and frequency in other pediatric chronic pain conditions [16],[17] with emerging evidence showing improvement in pain-related functional disability as well [17],[18]. The addition of biofeedback to relaxation training does not necessarily result in superior pain outcomes [17]. Modest data support the use of CBT in adults with chronic pain and disability [19] (see Box 1).

Psychological interventions are useful for the management of acute pediatric pain (see Box 1) although evidence for their efficacy varies depending on the type of pain. For procedure related pain, particularly needle procedures, distraction, hypnosis and CBT are effective, evidence based interventions [20]-[24].

In a review of non-pharmacological treatments to reduce the distress due to acute wound care in burn patients, some evidence has been shown that healthcare provider interventions (massage and maximizing patient control and predictability), child interventions (for example, virtual reality gaming [25] and therapist coaching of stress management techniques [26]) are beneficial.

For postoperative pain, there is insufficient evidence for the efficacy of most psychological interventions, but preparation, guided imagery and CBT are promising [27].

#### Psychological interventions for itch

Patients with EB may experience severe itch, which not only intensifies their suffering but can also be a source of social embarrassment secondary to the appearance of the excoriated skin and the preoccupation with scratching in social situations. Beyond this, damage of the integrity of the skin can create a portal of entry for systemic infections. Regardless of the underlying cause, itch evokes the behavior of scratching that increases inflammation and stimulates nerve fibers, leading to more itching and scratching [28]. Perpetuation of the itch-scratch cycle alters the integrity of skin leading to barrier damage. Scratching also causes undesirable changes in skin, such as lichenification and prurigo nodule formation. Successful treatment of itch requires interruption of this cycle. Non-pharmacological treatments, such as CBT, hypnosis, meditation, prayer, biofeedback and eye movement desensitization and reprocessing (EMDR), have been used with some success in both adults and children with conditions such as atopic dermatitis [29]. Habit reversal is a specific behavioral intervention whereby habitual behaviors are brought into conscious awareness and specific behavioral techniques are developed as a competing response to the urge to itch [30],[31] (see Box 1). In practice, this technique is frequently combined with CBT and relaxation as part of a treatment package applicable to patients of all ages [29],[32].

#### Integrative medicine therapies

Complementary and alternative medicine (CAM) practices constitute therapies that are often requested by patients and families for a variety of chronic pain conditions. This class of treatments includes, but is not limited to: acupuncture, meditation, massage, herbal preparations, yoga and chiropractic work. The evidence for therapies such as acupuncture [33], music therapy [34], chiropractic care, or yoga [35] is modest in the pediatric and adult pain populations, with no specific studies in EB. *Prima facie* evidence would suggest that massage and chiropractic interventions might be harmful given the skin fragility and the osteopenia that is often seen in EB. With regard to herbal supplementation, one of the chief side effects for a number of these preparations is bleeding, although the evidence is not clear [36]. Thus, this treatment may be contraindicated in patients having surgery, open wounds or a history of gastrointestinal tract bleeding. The active ingredients in the herbal preparations may also have interactions with any other prescribed medication; attention to potential drug-drug interactions should be considered.

##### Good practice points

Assessment of the suitability of psychological therapies for EB patients experiencing chronic and acute pain should always take into account developmental issues including age, cognitive level and psychopathology [37]. Include parents in behavioral pain management interventions [38] for children and adolescents.

### Acute pain care: postoperative pain management

#### Introduction

As a multisystem disorder, EB causes a variety of disruptions to body systems that are amenable to surgical intervention. While there are no controlled trials of postoperative pain therapies in EB, general principles of pain care apply.

#### Assessment

As for all patients, pain should be regularly assessed, and then reassessed after intervention to evaluate analgesic efficacy and detect side effects. Numeric rating scales have been shown to be effective for children of a developmental level above eight years who can verbalize their pain scores as well as for adults. Pain assessment in younger or nonverbal patients can be completed using the Face Legs Arms Cry Consolability (FLACC) and Children’s Hospital of Eastern Ontario Pain Scale (CHEOPS) [39],[40] augmented by reports of parents or other close family care-givers.

#### Systemic therapies

Analgesic treatment starts before and during surgery and includes the use of opioids, non-steroidal anti-inflammatory drugs, acetaminophen and, when appropriate for the type of surgery, regional anesthesia [41],[42]. Patient controlled analgesia (PCA) technology is a safe and useful way to deliver opioids to patents of all ages with EB, as it is for non-EB patients (see Box 2).

In the postoperative period, EB patients may be slow to resume oral intake, especially after oropharyngeal procedures, such as esophageal dilatation or dental rehabilitation. For patients lacking gastrostomy tubes, intravenous analgesia may be required. Intravenous analgesia should be multimodal and may include opioids (for example, morphine or hydromorphone), nonsteroidal anti-inflammatory drugs (NSAIDS; when postoperative bleeding is not a risk and renal function is normal (for example, ketorolac)) and acetaminophen (enteral or intravenous). Given the high rate of superficial bleeding from skin wounds, cyclooxygenase-2 inhibitors may have a role to play, given they have prothrombotic features in some studies [43]. Due to the risk of blister formation with rectal manipulation, there is controversy regarding whether the rectal route of administration may be used in EB patients [44]. Intranasal opioids can be effective for short-term treatment, if other routes are not available [45]-[47], and may be particularly helpful for procedures of short duration when rapid-acting analgesia is desired in the absence of intravenous access. Oral disintegrating and trans-buccal formulations may be helpful as well, although they presume intact mucosa, which may not be applicable (see Box 2).

Patients with prolonged postoperative pain because of extensive surgery may benefit from the use of opioid-based PCA with dose adjustments that take into consideration prior opioid use. While no EB-specific literature exists, this modality is standard treatment in many centers for patients old enough to understand the concept (typically seven years of age or older). Activation of the dosing button may be limited by pseudosyndactyly, so use should be determined on a case-by-case basis. PCA-by-proxy (dose administered by a nurse or designated family member) is practiced in some pediatric centers and may be an alternative approach to PCA dosing for children.

#### Preoperative tolerance

The preoperative pain status of the patient should be taken into consideration when choosing opioid dose, both intra- and post-operatively. Many EB patients have recurring acute and/or chronic pain and may be taking opioids daily, either prior to bathing/dressing changes or for ongoing pain. These patients require increased doses of opioids for adequate analgesic effect, due to the development of tolerance. Their usual daily opioid intake should be considered the baseline opioid dose to which the postoperative analgesia should be added (see Box 2). Drug pharmacokinetics may be altered in patients chronically exposed to sedatives and opioids. Flexibility in dosing, review of prior anesthetic records and discussion with the patient or family can aid appropriate dosing.

#### Regional anesthesia

Some common procedures (and the corresponding regional anesthesia technique used) in EB patients include: fundoplication (epidural) [48]-[50], hand and foot surgery (peripheral nerve block, either single shot or continuous peripheral nerve catheter infusion following bolus injection) and hand surgery (both axillary and infraclavicular approaches have been used for brachial plexus block) [51],[52].

Regional anesthesia techniques should be modified in patients with EB to minimize damage to the skin. Skin preparation may be performed by pouring prep solution (povidone-iodine or chlorhexidine) on the site without rubbing, then blotting and allowing the solution to dry. Ultrasound guidance can be used for peripheral nerve blocks with generous use of gel to minimize skin abrasion when moving the probe. When a catheter is placed (epidural or peripheral nerve), it may be tunnelled subcutaneously if desired [53] then secured to the skin using silicone-based tapes and dressings (Mepitac®, Mepitel®, Mepilex®) or other soft non-adhesive dressings. Adhesive dressings and adhesive adjuncts should be avoided. If there is any doubt about the safety of using a dressing, it should be tested on a small area of skin with patient or parental consent. With local infection being a relative contraindication to regional anesthesia, examination of the skin overlying the point of entry is mandatory prior to attempting a catheter placement. If leaving a catheter in the epidural space is undesirable, then a single injection of a long-acting form of opioid (DepoDur®) may be a good option when available [54],[55], although monitoring is important due to risk of hypoventilation [56]. Lidocaine infiltration may be used by dentists/oral surgeons when extractions are necessary, but care must be taken when injecting to avoid mucosal blister formation (see Box 2).

#### Non-pharmacologic therapies

Non-pharmacologic techniques should also be employed in combination with the pharmacologic techniques listed above. These include, but are not limited to, distraction (music, reading, video games and movies), visualization, imagery/virtual reality and breathing techniques (see Efficacy of Psychological Interventions in Chronic and Acute Pain Management, above and Box 1). Effective approaches used in the past should be discussed with the patient or parents and their use supported whenever possible.

##### Good practice points

Postoperative pain can be managed as for other patients in the same setting, with modifications to account for the ability to self-administer medications, prior drug exposure, and the condition of the skin at potential regional anesthesia injection/catheter sites.

### Chronic and recurrent pain care

EB has a large number of painful complications, some chronic and others acute but repetitive. Care of EB involves painful interventions on a daily basis. Almost every organ system can be affected, but the following are the major sources of pain: skin, gastrointestinal tract, musculoskeletal system, eyes.

#### Skin and wound pain

##### Introduction

The classic presentation of EB is the development of skin wounds that are painful in their own right, but which often become infected, heal poorly and frequently lead to scarring. This combination makes skin and wound pain a prominent complaint of patients with EB.

##### Environmental and behavioral approaches

Dressings that are non-adhesive upon removal, such as silicone-based products, are helpful in reducing skin-trauma pain. Different subtypes of EB and different individuals seem to have varying dressing requirements [57]. Nutrition is important in promoting wound healing, and anecdotally, patients with poor nutritional status have more wounds and slower healing, which lead to increased pain. Experience suggests that attention to good nutrition, surveillance for superficial infection and aggressive treatment of infection reduce pain. As discussed in the section on psychological interventions, CBT is helpful for a number of painful conditions and should be applied in EB.

##### Systemic approaches

The pharmacological treatment of skin and wound pain is non-specific, with no evidence in the EB population to promote one treatment over another. NSAIDs, acetaminophen, tramadol and opioids are used with success. Cannabinoids have some anecdotal support. Itch is a major problem (see below) that can lead to increased scratching and subsequent development of painful wounds. Hence, managing pruritus is important to prevent wound-related pain.

Many patients with severe types of EB use long-acting opioid preparations to provide a basal comfort level. Of note, there are no clear guidelines that long-acting opioids are preferable to use of intermittent opioids for non-cancer pain [58], although there is modest evidence for opioid use, in general, for non-cancer pain in adults [58],[59]. Individualization of therapy is recommended.

Regular and frequent dosing of opioids is common and merits special attention. Regardless of the opioid chosen, chronic use of opioids may have endocrinologic effects, such as hypogonadism [60]. Monitoring should be considered, especially given the propensity for osteopenia and delayed puberty in adolescents and young adults with EB. In the absence of data, the use of opioids is recommended as clinically indicated (see Box 3).

Methadone merits particular consideration. Dose titration can be unpredictable due to long and variable half-life as well as to saturable plasma protein binding [61]. The long and variable half-life, as well as the risk of methadone-induced prolonged QT syndrome [62],[63], mandates care in its use alone and with other drugs that may also prolong the QT interval. Guidelines on electrocardiographic screening are limited [64] (especially for adult patients of smaller stature and children). Given the risk of prolonged QT and methadone’s complex and variable pharmacokinetics, it is recommended that methadone only be prescribed by practitioners with experience evaluating and monitoring its use [65]. Methadone use is, therefore, best done with input from a pain management and/or palliative care specialist.

Among the common side effects of opioids, constipation [66] and pruritus are particularly significant. Both are significant baseline problems in EB and the reader is referred to the sections on gastrointestinal pain and pruritus.

The pain of extensive wounds has a quality suggestive of neuropathic pain; both are often described as `burning’ in quality. There is evidence supporting the use of gabapentin for neuropathic pain in other conditions [67]. Medications such as tricyclic antidepressants and gabapentin have shown anecdotal success in EB skin pain in a child [68], as well as for adult patients with acute skin pain due to burns [69]. The potential for tricyclic antidepressants to prolong the QT interval means that caution should be taken using this class of medications in RDEB patients, who are at risk for developing cardiomyopathy [70], as a lethal outcome in this setting has been reported [71].

##### Topical approaches

For localized wounds, topical lidocaine jelly (2% concentration) has been used anecdotally. Morphine mixed in hydrogel formulations has been used in different types of localized painful wounds [72],[73] but has only limited anecdotal experience in EB wounds [74] (see Box 3). Use of these medication vehicles raises questions of absorption and systemic effects that require further investigation before full recommendations can be made. Limited anecdotal evidence supports the use of keratinocyte hydrogel dressings for poorly healing wounds and RDEB [75].

Blood draws and intravenous access as well as the need for skin biopsies are minor technical procedures that can be very distressing, especially for children. Topical anesthetics are commonly used in non-EB populations with good effect. Amethocaine appears to be more effective than a eutectic mixture of local anesthetics (EMLA®) [76]. These may have a role when applied to intact skin, but cannot be recommended for use on wounds due to unknown rate and extent of absorption of lidocaine. Blistering as a localized allergic reaction to EMLA® (or its contents) has been rarely observed, indicating caution in its use even on intact skin. Lidocaine injected with a small gauge needle is a standard approach to superficial analgesia in all age groups. Additionally, it is recommended that bicarbonate be added to buffer the pH of the lidocaine to reduce the pain of injection [77]. As noted above, CBT (for example, distraction and relaxation) is effective in the acute pain setting [21] and should be employed whenever possible in conjunction with medication management (see Box 1).

##### Good practice points

Prevention and rapid healing of wounds through non-adherent dressings, optimal nutrition and infection control are priorities.

#### Bathing and dressing changes

##### Introduction

Bathing and bandage changes are a source of significant recurrent pain and anxiety for patients with EB.

##### Environmental treatment

Oatmeal and salt have been added to bath water to reduce the pain of immersion [78]. The former may have anti-pruritic, cleansing and protective properties for dermatologic conditions in general [79] that may be similarly effective in EB. Salt is added to make the water isotonic, which has anecdotal support from many families of patients with EB including preliminary survey data from EB families [80]. Isotonic saline (0.9% NaCl) is 9 grams of salt/1 liter of water; total amount of salt varying according to the desired water volume. Bleach and vinegar are anecdotally helpful in drying the skin and reducing bacterial colonization, which some patients find comforting while others report increased pain at the wound sites. The salt water does not require plain water rinse, whereas vinegar and bleach baths require rinsing to reduce the risk of itch. Many patients elect to reduce the frequency of bathing as a simple measure to minimize bath-related pain. Anecdotally, starting the bath by immersing the patient still in his/her bandages can ease the transition into the water and help reduce the pain of removing the dressings. Other patients find drafts from heating and cooling systems to be painful on open wounds, so attention to general environmental factors is recommended (see Box 4).

##### Analgesics

Analgesics used by patients with EB have included enteral opioids, NSAIDs and acetaminophen. The nasal route may permit administration of medications when intravenous access is absent [81]. Fentanyl works well by this route for non-EB patients in the perioperative setting [45], for breakthrough cancer pain (see section on Breakthrough pain medication at the end of life) and has been used for acute pain management in the emergency department with success [46]. Butorphanol is a mixed agonist-antagonist that can be used via the intranasal route and is effective both for pain (for example, dental postoperative pain [47]) as well as for opioid-induced pruritus. Fixed dose spray is available for outpatient use in the United States, which limits the ability to adjust for patient size. Timing of medication administration should be adjusted to match expected peak analgesia with anticipated peak pain (see Box 4).

Ketamine can be administered enterally for wound care pain, for which there are data from adult and pediatric burn populations [82],[83] but not for EB patients. The IV form of ketamine given orally has been used with variable success for other chronic pain conditions [84] as well as in cancer patients [85] and in pediatric palliative care [86].

Inhaled nitrous oxide has been suggested for pediatric wound care in EB in some texts and review articles [44],[87], but its use in EB patients has not been studied or reported. Nitrous oxide has been used for painful procedures in other settings [88] and is available in both fixed 50:50 and variable combinations with oxygen. Depending on the delivery system (mouth piece, face mask, or nasal mask) concerns arise about environmental pollution and exposure of health care personnel [89]. In the absence of evidence of efficacy, concern must be raised about repeated use for wound care or dressing changes because of longstanding evidence of megaloblastic bone marrow changes and neurologic abnormalities due to inhibition of methionine synthase and cobalamin activity with repeated exposure to nitrous oxide [90],[91].

##### Anxiolysis

The pain caused by baths and dressing changes is commonly cited by families as generating anxiety in the patient, which in turn is stressful for the caregiver. Medications used for acute anxiolysis in this population include midazolam, diazepam and lorazepam and are considered good treatment for acute anxiety in other populations (for example, children for dental procedures, [92],[93]; prior to adult burn procedures [94]). In addition to anxiolysis, the pre-procedural use of benzodiazepines offers the possibility of anterograde amnesia which may prevent the development of increased anxiety with repeated procedures. When using benzodiazepines in combination with opioids, care must be taken to avoid over sedation (see Box 4). As response to each medication will have individual variation, dose adjustments should be made carefully and preferably one drug at a time, when more than one is being administered.

##### Other behavioral comfort measures

In addition to medication management, recommendations from caregivers include preparing all materials ahead of bandage removal to reduce the amount of time wounds are exposed to air. Involving children in the process at as early an age as possible helps them to develop a sense of control. Maintaining room temperature at a moderately warm level has also been recommended. CBT can be applied to treating the anxiety and pain associated with bathing and bandage changes (see section on psychological approaches, above).

##### Good practice points

Bathing and dressing changes are recurrent sources of both pain and anxiety. Therapy should focus on both symptoms, using environmental, psychological and pharmacologic approaches.

#### Pain related to the gastrointestinal tract

##### Introduction

Gastrointestinal complications are common in children and adults with EB with specific complications linked to different subtypes [95]-[97].

#### Upper gastrointestinal tract

##### Topical treatments

Oral ulceration, blistering and mucositis are the most frequent gastrointestinal complications in patients with EB [98]. While oral ulceration can occur in all types of EB, it is most common and problematic in patients with the severe types of this disease. Oral ulceration can be extremely painful and lead to difficulties in feeding and maintaining dental hygiene. Apart from oral analgesics, topical preparations are available. Sucralfate suspension has been used to prevent and manage oral blisters in five children, 6- to 11-years-old, with RDEB when applied four times a day for six months [99]. The number of blisters reduced within three weeks and pain reduced within one week (see Box 5).

Polyvinylpyrrolidone-sodium hyaluronate gel (Gelclair®) either used as a mouth rinse or applied directly to a mouth lesion in children, has been used anecdotally in EB. Secondary evidence comes from two recent reviews which showed that Gelclair® may be useful as an adjunctive therapy in decreasing the painful lesions of oral mucositis in cancer patients [100],[101]. Chlorhexidine gluconate and benzydamine hydrochloride mouth spray is used regularly in EB patients at the London centers with reported benefit but has not been evaluated systematically. Secondary evidence for the potential efficacy of this intervention exists for treating the pain of acute viral pharyngitis in adults [102].

##### Gastroesophageal reflux

Patients with EB are at high risk for gastroesophageal reflux disease (GERD) - especially infants with generalized EBS, JEB and patients with severe generalized RDEB who can develop esophagitis [95]. While children with GERD may not complain of dyspeptic symptoms until they are older, crying and sleep disturbance are commonly associated with this symptom. Management is guided by standard treatment in non-EB patients, and may involve treating the reflux with antacids, histamine H_2_-blockers and proton pump inhibitors and, rarely, surgery [103],[104] (see Box 5).

##### Esophageal strictures

Esophageal stricture formation and dysphagia are most common in patients with severe generalized RDEB with a cumulative risk of developing strictures or stenosis rising steadily from 6.73% at age 1 year to 94.72% at age 45 years [105]. However, strictures can also be seen in other subtypes of EB including Kindler syndrome [106]. Dysphagia and delayed progression of food through the stricture can present with retrosternal chest pain. Esophageal strictures, if symptomatic, require treatment with a fluoroscopically guided balloon dilatation with peri- and post-operative steroids administered to reduce the recurrent stenosis rate [107]-[109] (see Box 5). Acute symptoms can respond temporarily to oral dexamethasone or nebulized budesonide, based on anecdotal evidence. A recent case report examined the use of daily oral viscous budesonide therapy (0.5 mg/2 mL budesonide nebulizer solution mixed with 5 g of sucralose and maltodextrin) in two children with RDEB who had recurrent proximal esophageal strictures [110]. Both children had a reduction in the rate of stricture formation and symptoms of dysphagia.

#### Lower gastrointestinal tract

##### Constipation

Constipation has been shown to be present in 35% of children with all types of EB [95] and can cause abdominal pain and pain on defecation as well as lead to perianal trauma. A case of colonic perforation and early death resulting from severe constipation in DEB has been described [111]. Chronic perianal pain during defecation can produce stool withholding behavior which exacerbates constipation. Chronic opioid use may contribute to poor bowel motility. First line management is prevention with dietary manipulation; however, patients with severe types of EB often require the regular use of a laxative (see Box 5). Polyethylene glycol is effective clinically in treating constipation in children and adults with EB, with empiric evidence of efficacy in adult [112] and pediatric [113] non-EB cohorts.

##### Colitis

A subgroup of children with RDEB may develop colitis, presenting with abdominal pain and other symptoms [95],[114]. Dietary restriction and anti-inflammatory drugs, such as sulfasalazine, have been used but with variable effects on controlling the colitis [95].

##### Perianal pain

Perianal blistering and ulceration is common and debilitating in children and adults with severe types of EB. Topically, sucralfate has been shown to be superior to petroleum jelly in facilitating anal fistulotomy healing and lessening wound pain [115]. Sucralfate suspension has also been used to lessen the pain of oral and genital ulceration in patients with Behcet's disease [116]. Anecdotally, topical sucralfate combined with Cavilon™ (an alcohol-free liquid barrier film that dries quickly to form a breathable, transparent coating on the skin, containing hexamethyldisiloxane, isooctane, acrylate terpolymer, polyphenylmethylsiloxane) appears to improve pain in perianal lesions of some patients with EB. Non-surgical treatment is of small benefit for both pain and healing of chronic anal fissures [117], with recommendations of topical diltiazem and glyceryl trinitrate followed by surgery if needed in adults [118]. No data on anal fissure treatment in EB patients exist (see Box 5).

##### Good practice points

EB affects the gastrointestinal tract in its entirety. Pain that originates from ulcerative lesions may respond to topical therapy. GERD and esophageal strictures have nutritional as well as comfort implications and should be addressed promptly when found. Maintaining good bowel habits and reducing iatrogenic causes of constipation are crucial.

#### Musculoskeletal pain

##### Introduction

Musculoskeletal complications are common and frequently painful for patients with EB. These conditions include pseudosyndactyly, osteopenia, back pain, fractures and occasional co-morbid rheumatologic disorders.

##### Joint pain

Pseudosyndactyly affects hands, feet, ankles and wrists. Painful hyperkeratotic lesions develop on the soles of patients with EBS. Occupational therapy approaches can improve function and decrease pain via use of adaptive equipment and specially designed orthotics and clothing. Physical and occupational therapy play crucial roles in encouraging patient mobility. For weight-bearing patients, careful attention to footwear, nails, orthotics and hyperkeratosis management are important aspects of pain care [119]. Referring patients to programs to maintain or regain strength, to prevent or minimize joint contractures, and to optimize mobility is recommended based on anecdotal evidence (see Box 6). Coping skills training and activity pacing can enhance effectiveness of therapy [120].Surgical intervention has had some success in increasing mobility and reducing pain [121]. EB patients can also have inflammatory arthritis [122],[123]; appropriate imaging can be helpful in determining inflammatory causes of pain. Given that inflammatory markers are usually elevated in EB patients, these markers alone are not likely to be useful to diagnose joint pathology.

##### Bone pain

Osteopenia, osteoporosis and fractures are now well recognized and commonly seen in patients with the severest types of EB [124]-[126], as a cause of bone and joint pain. The causes are multifactorial and include reduced mobility, delayed puberty, limited exposure of the skin to sunlight, inadequate nutritional intake for metabolic requirements and chronic inflammation. Chronic inflammation causes increased osteoclast activity [127],[128].

As a consequence of the osteoporosis, a significant proportion of patients develop fractures. The incidence of vertebral fractures in patients with RDEB is unknown, but may be underestimated due to the fact that fractures of the lumbar and thoracic spine may be clinically silent, or present without overt or localized back pain on palpation [125]. Anecdotally, however, some patients can present with back pain, in which case a high index of suspicion should be maintained. Treatment of osteoporosis and derivative pain is based upon standard treatment of osteoporosis in non-EB patients, which relies on vitamin D and calcium supplementation, exercise and bisphosphonate treatment [129] (see Box 6).

Anecdotally, bisphosphonates have been found useful in treating patients with evidence of osteoporosis without fracture. Bisphosphonates appear to improve fractures on annual X-rays and lead to a noticeable improvement in pain. Indirect support for the use of bisphosphonates comes from its use in rheumatologic and other pediatric conditions, associated with osteopenia [130],[131] and resolution of back pain [131],[132], but efficacy specifically in patients with EB has not been formally studied.

##### Back pain

Older patients with EB can have back pain. Beyond osteopenia-related issues, biomechanical causes can be involved. Foot blisters and painful hyperkeratosis can cause abnormal gait and compensatory postures. Further, impaired mobility can add a myofascial component to pain. Management of back pain includes optimal foot care, assessment of primary and secondary mechanical factors, evaluation and treatment of osteopenia and fractures, physical therapy, standard analgesics and CBT interventions. Back pain treatment in the EB population is extrapolated from basic principles recommended for the general population [133], for lack of EB-specific evidence (see Box 6).

NSAIDs are routinely used for bone and joint pain of a variety of types and are appropriate for these types of pain in EB. Care should be taken to monitor for side effects, and the cyclooxygenase-2 inhibitors may be useful in those patients who experience improved pain control but note increasing blood loss from wounds, or who have gastrointestinal upset with standard NSAIDs. Acetaminophen does not affect bleeding and can be useful. Tramadol and opioids are also useful for more severe pain. Some prefer use of long-acting opioid preparations, with methadone being the only one available in liquid form. Limitations in methadone use are discussed in sub-section on systemic approaches under Skin and Wound Pain.

##### Good practice points

Bone health should be optimized with calcium and vitamin D supplements as needed, maintenance of maximum mobility, minimization of joint deformity and monitoring for/treatment of pubertal delay. Routine screening for bone mineral density may be useful to ascertain cases of osteopenia before they progress to osteoporosis and fractures. Care must be taken not to overlook inflammatory joint disease, as pain secondary to this will respond to disease modifying therapy. Multidisciplinary treatment modalities are helpful in addressing back pain and apply to such pain in the EB population as well.

### Eye pain

Ophthalmologic involvement is pervasive in EB [134],[135]. Eye pain is often caused by corneal abrasions. Comfort measures such as avoiding bright light (a natural reaction to the photophobia that results from the trauma), lubricating eye drops, NSAIDs and antibiotic drops have been used. Evidence for these treatments for recurrent corneal abrasions of other etiologies is modest [136]. The use of eye patches in non-EB patients does not aid pain relief and, therefore, is not recommended for use in EB patients either [137]. There is some evidence that topical NSAIDs provide analgesia for acute, traumatic corneal abrasions [138] (see Box 7).

#### Good practice points

Corneal abrasions are painful and common in EB; prevention and supportive care are appropriate.

### Special Topics

#### Pain care in infants with EB

##### Introduction

In the severe forms of EB, pain starts immediately, and great care needs to be taken in most of the activities of daily living. Infants with EB require careful attention to environmental factors at a higher level than for older children but otherwise can receive pain care using guidelines for specific types of discomfort.

##### Pain assessment

Pain assessment should be completed on all neonates with EB at regular intervals and as needed (see Box 8). A valid neonatal pain scale should be used, considering both physiologic and behavioral measures. Some examples include: the Premature Infant Pain Profile (PIPP) [139],[140], the Neonatal Pain Agitation and Sedation Scale (N-PASS) [141], the Crying, Requires oxygen, Increased vital signs, Expression and Sleepless pain scale (CRIES) [142], and the Neonatal Infant Pain Scale (NIPS) [143]. The Face Legs Arms Cry Consolability (FLACC) scale is recommended for infants between 1 to 12 months [144]. These pain scales have been validated for use by nurses, although training could be generalized to non-nurse care givers and family members. Pain should be reassessed frequently during and after interventions and until comfort is achieved based on a combination of one of the above scales and clinical judgment. Analgesic interventions should match degree or level of pain. Of note, infants, especially neonates, seem to be a higher risk for respiratory compromise [145],[146] due to reduced clearance of opioids in infants younger than two months [147],[148]. Therefore, careful clinical and cardiopulmonary monitoring is crucial when providing opioids to this population (see Box 8).

##### Procedural pain

As for older patients, procedures are one of the major sources of pain for infants with EB. Wound care specifically requires a multidisciplinary team approach to ensure consistent and optimized pain control. There is evidence that a variety of pharmacologic and non-pharmacologic interventions are helpful in both term and pre-term infants [149]. Therefore, pain management interventions should precede all scheduled painful procedures, such as dressing changes, bathing and venipuncture. The range of analgesics and the routes by which they can be administered are the same as for older patients. Oral sucrose is an analgesic unique to young infants and can be used alone for mild pain (for example, immunization pain [150]) or in conjunction with other analgesics as well as physical and environmental interventions for more severe acute pain [40],[151].

##### Bathing and dressing changes

To maintain appropriate moisture levels and facilitate the healing of wounds, dressings should be non-adherent as for older patients and appropriate in size. Infected wounds will require more frequent dressing changes [57],[152]. Changing dressings one limb at a time is advocated in infants, to reduce the likelihood of having the skin rubbed off the opposite limb by feet kicking together, causing new, painful lesions (see Box 8). This approach also decreases the chance of bacterial spread from a colonized wound to an uncontaminated area [57],[78]. For baths, adding salt to the water is recommended as for older patients (see subsection on environmental treatment under Bathing and Dressing Changes).

In addition to environmental adjustments, many infants will require analgesics with bath and dressing changes. NSAIDs, acetaminophen and opioids are all used, as for older patients (see Section-Bathing and Dressing Changes) (see Box 8). Codeine is not recommended, when alternatives are available, as neonates lack the capacity to metabolize the drug into active metabolites, and clinical responses are highly variable at all ages, due to polymorphisms in codeine’s metabolic pathway [153],[154].

##### Severely affected hospitalized infants

Severely affected newborns with deep tissue damage may require extensive pharmacologic support to achieve a level of comfort (see Box 8). These infants may require around the clock or continuous infusion of opioids and an adjuvant. A transition to methadone for better steady state levels should be considered (refer to sub-section on systemic approaches under Skin and Wound Pain) for caveats about methadone use). A case study has shown effective pain control in an infant with severe chronic pain (and possibly pruritus) when treated with gabapentin [155]. Patients receiving frequent opioid treatment may require extra care in avoiding opioid-induced constipation and in maintaining adequate caloric intake.

Oral ketamine has been used to supplement opioids when pain associated with dressing changes is severe [156]. Use of ketamine raises concerns due to findings of adverse neurodevelopmental effects in young animals who received prolonged intravenous infusions of ketamine [157]. However, these effects are not known to occur in human infants and are unlikely to occur with small doses administered at intervals.

##### Good practice points

Infants benefit from the same range of analgesics as older patients. They can uniquely benefit from oral sucrose for brief painful episodes. Careful monitoring is crucial in infants receiving sedating medications. Babies with EB and their parents benefit from close physical contact like any other families. Holding and cuddling can be done safely, with scrupulous attention to lifting and handling in order to prevent new lesions and pain.

#### End of life pain care

##### Introduction

The epidemiology of death in EB varies by type [158], but is not limited to dermatologic causes. Patients with JEB, generalized severe type are at greatest risk of fatality early in life from a range of causes [159],[160]. Persons with RDEB may succumb in early to mid-adulthood from multiple causes [161]-[163] whereas patients with generalized types of EB simplex are at risk of laryngotracheal complications [164].

The palliative approach to pain experienced by individuals who are facing life-threatening illness addresses the physical, emotional and spiritual suffering of the patient and includes support for the whole family [165]-[167] (see Box 9). Squamous cell carcinoma is frequently diagnosed in patients with severe subtypes [168] and can present challenges typical of cancer-related pain when it metastasizes. The World Health Organization ladder approach to pain care for those patients with cancer and other persistent illnesses recommends a two-step `ladder’ as a framework for pain care [169].

##### Opioids

Opioids have a role in EB pain management, as previously indicated in these guidelines (see Box 9). Patients in the palliative phase of their illness may be given an oral opioid sustained release medication [170]. Opioids should be titrated against pain and side effects, with differences in approach needed depending on issues such as opioid tolerance and rate of development of new pain problems [170]. However, if appropriate opioid increases are not effective, it is important to consider pharmacologic tolerance, poor absorption and neuropathic pain. Tolerance can be addressed by rotating the opioid to capitalize upon incomplete cross-tolerance [171],[172]. In the face of poor absorption, parenteral therapy may be needed (see below). If neuropathic pain is suspected, methadone may be an option to consider. It has N-methyl-D-aspartate (NMDA) antagonist action in addition to mu-agonist properties common to standard opioids, and can be beneficial for neuropathic pain [173] and does not rely on renal excretion [61], which may be reduced in the palliative phase of EB [87]. Although a recent meta-analysis could not establish differences in efficacy among types of opioids for neuropathic pain, it was determined that intermediate term (weeks to months) use of opioids for neuropathic pain may be helpful [174]. Cautions in the use of methadone are discussed in the sub-section on systemic approaches under Skin and Wound Pain.

##### Adjunctive measures for neuropathic pain

Many EB patients will have been treated with adjunctive agents such as amitriptyline or gabapentin earlier in their illness, often with good effect [87],[155]. These both may take a period of time to achieve full analgesic effect and usually need to be given by the enteral route. Ketamine is more rapidly effective and can be given orally [84]-[86],[175] or used subcutaneously (see below). It should be noted that when ketamine by-passes hepatic first pass metabolism, the relative proportion of norketamine is reduced and the patient may experience more sedation and hallucinations and less analgesia for a given dose [175]. Other options for addressing neuropathic pain (see Box 9) in terminal care include opioid rotation to methadone (see above), which can also be included in a subcutaneous infusion (see below).

##### Infusions and issues of drug delivery

Parenteral administration of analgesia may be needed in the palliative phase, when ability to take enteral medications is no longer an option or if rapid escalation of therapy is needed. There has been an historic reluctance to use continuous subcutaneous infusions, due to the fear of precipitating further blistering. However, subcutaneous infusions have been reported to be tolerated in the final days of life in an adult EB patient [176] (see Box 9).

In non-EB patients with cancer pain, the subcutaneous route of morphine is effective to a similar degree as the intravenous [177]. Other alternatives, such as adherent transdermal delivery systems, need regular removal and replacement. Repeated subcutaneous/intramuscular injections are very frightening for children, as are attempts to obtain IV access. Alternatively, sites of subcutaneous needle insertion last reasonably well [87],[177]. If subcutaneous sites do become inflamed, some benefit has been shown from adding low dose dexamethasone to the infusion [178], although this has not been used to our knowledge in children with EB.

##### Breakthrough pain medication at the end of life

Even when good baseline pain relief has been achieved, most patients will require occasional `breakthrough’ doses of analgesia. Uniformly effective breakthrough dosing protocols have not been determined for children with EB at end of life, although there are general recommendations from several international sources [169],[179] and data to support the use of up to 20% of total daily opioid consumption [180],[181] in adults with cancer. In general, a short-acting, immediate release form of opioid should be given at approximately 10% to 15% of the total background opioid dose of the previous 24 hours. If rescue dosing is used frequently and consistently, then the baseline opioid dosing needs to be increased. Individual patient factors should be taken into consideration (including prior opioid exposure, renal and hepatic function, other medication use) and consultation with an expert in pain management and/or palliative care is recommended. The same principle is recommended for opioids given via all routes of administration. When very rapid onset is needed, transmucosal administration of opioids may be preferable (see Box 9). Commercially available transmucosal preparations of fentanyl are available in doses likely to be suitable for older children and adults. Evidence supports the use of transbuccal fentanyl for breakthrough pain in cancer patients [182]. An additional benefit of fentanyl is that it is not dependent on renal excretion. In some centers, the injectable formulations of morphine or diamorphine have been used buccally at around a third of the standard enteral dose (equivalent to the intravenous dose), allowing more flexibility of dosing. It is also important to remember that extreme anxiety may confound pain perception or expression and that there may be benefit in offering additional doses of buccal midazolam where this is felt to be a significant factor. Therapy should be targeted at specific symptoms whenever possible, although combination therapy is not unusual. Breakthrough analgesia should be offered in the face of unexplained `agitation’ in case of undiagnosed pain, after which medication for primary agitation/anxiety may be considered. While it is likely that both of these would cause a degree of sedation at higher doses, this should not be the primary aim of treatment.

##### Good practice points

Palliative pain care is an expected part of care for EB, which in many cases is life-limiting in nature. All basic principles of palliative care apply as they do for other terminal disease states.

#### Pruritus

##### Introduction

Itch is a prominent, debilitating and damaging symptom for children with EB, but the mechanisms which lead to the manifestation of pruritus are poorly understood [183]. Promising new insights into the causes and potential treatments of chronic itch in animal models [184], in human chronic itch generally [185] and in recovering burn patients [186] may hold potential for application in the EB population.

##### Non-pharmacological approaches

Multiple environmental and behavioral approaches to address itch have been suggested [183],[187] and include: prevention of dry skin (systemic hydration and emollients), gentle debridement of dry/dead/crusted skin, prevention and treatment of skin wound infection; maintaining/supporting the healing process by attention to anemia and nutritional status, limiting damage to the skin by avoiding shear forces imposed by scratching (short nails, occlusive barriers, patting rather than pulling or tearing at a site), avoiding overheating and using measures to keep the body cool (see Box 10). CBT is useful to reduce habitual scratching behaviors (see section on psychological approaches). It is important to avoid and treat secondary causes when present (such as drugs, environmental itch triggers, underlying co-morbidities). Opioid-induced itch in EB can be a problem and a difficult side effect to balance against the desired analgesia; opioid rotation may be helpful.

##### Pharmacological therapies

Pharmacological therapies include traditional oral antihistaminic medications (see Box 10). Some patients prefer sedating formulations at bedtime (for example, diphenhydramine, hydroxyzine, chlorpheniramine) and others non-sedating preparations during the day (for example, cetirizine, loratadine and fexofenadine). Efficacy is variable and recommendations are based on anecdotal experience for lack of good evidence in EB and other dermatologic conditions. Centrally acting medications, such as gabapentin and pregabalin, have evidence for efficacy from the burn literature [186],[188],[189] and may have application in EB. Many of these medications have also been used successfully for neuropathic pain (see sub-section on adjunctive measures for neuropathic pain under End of Life Pain). Antidepressants with nortriptyline re-uptake inhibitory actions have been used for various pruritic conditions with some evidence for efficacy, with mirtazapine showing promise [190]-[193]. Low-dose cyclosporine has been used in a single case of dominant dystrophic EB to reduce generalized itching [185],[194]. Anecdotal success has been reported with ondansetron in EB; however, the same had been reported for pruritus from cholestatic jaundice, although controlled trials did not confirm efficacy [195]. Similar suggestions were made related to the pruritus of uremia [196],[197], leading to recommendations that ondansetron should be considered, although clinical trials are needed in EB.

Other medications that have shown sporadic clinical success in treating itch in EB include: cyproheptadine; selective serotonin re-uptake inhibitors (SSRIs; fluoxetine, paroxetine, sertraline, fluvoxamine) [193]; tricyclic antidepressants (doxepin, amitriptyline, nortriptyline) [192]; opioid antagonists (naloxone, naltrexone); kappa receptor agonists (butorphanol, dextromethorphan, nalbuphine); other 5-HT3 receptor antagonists (granisetron, dolasetron); antipsychotic agents (olanzapine, pimozide) and cannabinoids. These agents and the NK-1 receptor antagonist aprepitant may prove to be useful in certain subsets of patients, although evidence to guide therapy is lacking [198] and practitioners are encouraged to pursue the aforementioned therapies based on patient response to prior medications, potential interactions within the patient's medication list and medication availability (see Box 10).

It is common practice for medications from different drug classes to be used concurrently and for agents in the same class to be rotated due to the observation that a particular medication may have improved efficacy after intermittent drug holiday.

##### Good practice points

A combination of environmental, cognitive-behavioral and pharmacologic therapies is available for use for EB-related pruritus, which can be a severe symptom of the disease.

## Conclusions

These guidelines represent the initial effort to organize pain management for patients with EB based on existing evidence. While the guidelines were developed using current evidence and a rigorous evaluation process, there are limitations in the clinical use of the recommendations. The two broad areas of concern involve practical limitations of implementation and the level of available evidence. Tables 5 and 6 identify issues for implementation of the guidelines as well as general recommendations areas for research to enhance the level of evidence.

**Table 5:** **Implementation barriers**

1.	Availability of resources (for example, medications and equipment)
2.	Legal and social restrictions on the use of various medications and therapies.
3.	Limited and uneven distribution of knowledge and expertise
4.	International dissemination of guidelines and EB-related information to local care providers and families (includes translation and access to electronic and print media)

**Table 6:** **Areas of research**

**Psychological and integrative approaches:**	
1.	Test the efficacy of well-established cognitive behavioral interventions for acute and chronic pain management in EB.
2.	Develop EB-specific pain assessment measures for both acute and chronic pain.
3.	Evaluate the efficacy of cognitive behavioral therapy for EB-related pruritus
4.	Evaluate the role for Integrative Medicine techniques for the EB population.
**Acute pain:**	
1.	Improve the balance between analgesia and side effects specific to EB (for example, itching).
2.	Establish optimal treatment of needle-related pain.
3.	Define the role for ketamine and other non-opioid agents.
**Chronic and recurrent pain:**	
1.	Evaluate topical therapies including opioids, local anesthetics and NSAIDs.
2.	Determine optimal environmental interventions for bath and dressing changes including bath additives (salt, bleach, oatmeal).
3.	Define optimal perianal pain therapies.
4.	Clarify the role of bone density screening in preventing bone pain and fractures.
5.	Determine the role of topical NSAIDs in treatment of corneal abrasion pain.
6.	Explore the role for various physical and occupational therapy interventions for joint, bone and back pain.
**Infants:**	
1.	Validate observational pain scales in the setting of bandaged infants.
2.	Determine the safety and dosing of adjunct medications, such as gabapentin and topical agents.
**Pruritus:**	
1.	Establish the mechanisms of pruritus in EB and effective treatment thereof.
2.	Refine the management of opioid-exacerbated itch.
**End of life:**	
1.	Define how best to integrate palliative care into the overall care of patients with EB prior to end of life.
2.	Define optimal treatments for pain at the end of life.

## 5Box 1. Recommendations for use of cognitive behavioral therapies in EB

1. For chronic pain management use cognitive behavioral therapy (CBT). **(Grade: B)**
2. For acute pain management, offer the patient distraction, hypnosis, visualization, relaxation or other forms of CBT. **(Grade: B)**
3. Consider habit reversal training, and other psychological techniques for management of pruritus. **(Grade: C)**

## 6Box 2. Recommendations for acute pain care in EB

1. Basic perioperative pain assessment and treatments should be used as for non-EB patients, with modification. **(Grade: A)**
2. Transmucosal (including intranasal fentanyl and trans buccal opioids) should be considered for short procedures and pain of brief duration when intravenous and enteral routes are unavailable. **(Grade: B)**
3. Perioperative opioid use must take preoperative exposure into consideration, with appropriate dose increases to account for tolerance. **(Grade: B)**
4. Regional anesthesia is appropriate for pain resulting from a number of major surgeries. Dressing of catheters must be non-adhesive and monitored carefully. **(Grade: C)**

## 7Box 3. Recommendations for wound pain treatment

1. Maintain optimal nutrition and mobility and treat infections as indicated **(Grade: D)**
2. Consider topical therapies for pain **(Grade: C)**
3. Systemic pharmacologic therapy should be adapted to treat both acute and chronic forms of skin pain. **(Grade: B)**
4. Monitor potential long-term complications of chronically administered medications. **(Grade: C)**

## 8Box 4. Recommendations for bathing and dressing change pain treatment

1. Anxiolytics and analgesics should be used for procedural pain and fear. Care must be taken when combining such medications due to cumulative sedative effects **(Grade: B).**
2. Cognitive behavioral techniques should be implemented as the child becomes old enough to use them effectively. Specifically, distraction should be used for younger children **(Grade: B).**
3. Environmental comfort measures are recommended; these include adding salt to the water to make it isotonic and keeping the room warm **(Grade: B).**

## 9Box 5. Recommendations for gastrointestinal pain treatment

1. Topical treatments are recommended for oral and perianal pain. **(Grade: C)**
2. Therapy should be directed to manage gastroesophageal reflux and esophageal strictures using standard treatments. **(Grade: C)**
3. Constipation should be addressed nutritionally, with hydration and addition of fiber in the diet to keep stool soft, with stool softeners and by minimizing medication-induced dysmotility. **(Grade: C)**

## 10Box 6. Recommendations for musculoskeletal pain treatment

1. Joint pain should be treated with mechanical interventions, physical therapy, cognitive behavioral therapy and surgical correction **(Grade: C).**
2. Osteoporosis should be treated to reduce pain in EB **(Grade: D).**
3. Back pain should be addressed with standard multi-disciplinary care **(Grade: C).**

## 11Box 7. Recommendations for eye pain treatment

1. Care should include general supportive and analgesic care, protecting the eye from further damage and topical therapies **(Grade: C).**

## 12Box 8. Recommendations for infant pain care

1. Assess patients as needed and prior to and after interventions; health care workers should use validated measures. **(Grade: A)**
2. Sucrose solutions should be administered for mild to moderate pain alone or as an adjunct. **(Grade: B)**
3. Standard analgesics should be used in infants as in older patients with careful attention to dosing and monitoring. **(Grade: B)**

## 13Box 9. Recommendations for end of life pain treatment

1. Assess and manage physical, emotional and spiritual suffering of the patient, while providing support for the whole family. **(Grade: A)**
2. Opioids are the cornerstone of good analgesia in this setting. Opioid rotation may need to be considered to improve analgesia and reduce side effects, and adjuncts may need to be added. **(Grade: B)**
3. Consider targeted medication for neuropathic pain or when pain proves refractory to convention therapies. **(Grade: D)**
4. Continuous subcutaneous infusion of medication is an option when parenteral therapy is needed in the terminal phase. **(Grade: C)**
5. Where needed, breakthrough medication can be given transmucosally (oral or nasal) for rapid onset and avoidance of the enteral route. **(Grade: B)**

## 14Box 10. Recommendations for itch treatment

1. Use environmental and behavioral interventions for itch control. **(Grade: C)**
2. Antihistamines are recommended and can be chosen depending upon desirability of sedating effects. **(Grade: D)**
3. Gabapentin, pregabalin, TCA, serotonin norepinephrine reuptake inhibitors (SNRIs) and other non-traditional antipruritic agents should be strongly considered for itch treatment. **(Grade: C)**

## Authors' contributions

This work was initiated and led by KRG with active input from all authors. JG, EH, CL, ALJ, AEM, LGM and DSL participated in the review process and recommendation/guideline draft, see text for details. DSL led the systematic evidence analysis and provided methodological support and guidance. Multi-disciplinary clinician input was obtained as was input from patients and families early in the process (see Acknowledgments, below). The consensus meeting (see text) was attended in person or via Skype by KRG, ALJ, EH, JG, CL, LGM and BK (see Acknowledgements). All authors read and approved the final manuscript.

## Additional file

## Electronic supplementary material

Additional file 1: EB-specific articles that were excluded from use in making recommendations, with rationales.(DOC )

Below are the links to the authors’ original submitted files for images.

Authors’ original file for figure 1

## Abbreviations

CBT: cognitive behavioral therapy/techniques
CHEOPS: Children's Hospital of Eastern Ontario Pain Scale
CRIES: Crying, requires oxygen, Increased vital signs, Expression and Sleepless Pain Scale
DDEB: dominant dystrophic epidermolysis bullosa
DEB: dystrophic epidermolysis bullosa (dominant and recessive types)
EB: epidermolysis bullosa
EBS: epidermolysis bullosa simplex (basal and suprabasal types)
FLACC: Face Legs Arms Cry Consolability Pain Scale
GERD: gastroesophageal reflux disease
JEB: junctional epidermolysis bullosa (generalized and localized)
NIPS: Neonatal Infant Pain Scale
NMDA: N-methyl-d-aspartate
NSAIDs: non-steroidal anti-inflammatory drugs
N-PASS: Neonatal Pain Agitation and Sedation Scale
PCA: patient controlled analgesia
PIPP: Premature Infant Pain Profile
RDEB: recessive dystrophic epidermolysis bullosa
