# Supplementary material for: Pain care for patients with epidermolysis bullosa: best care practice guidelines
Source: BMC Med. 2014 Oct 9;12:178. doi: 10.1186/s12916-014-0178-2 (PMC4190576; doi:10.1186/s12916-014-0178-2)
Supplement: Supplementary file 1 — Additional file 1: EB-specific articles that were excluded from use in making recommendations, with rationales.(DOC ) [file 12916_2014_178_MOESM1_ESM.doc]

**Additional File 1.** EB-specific articles that were excluded from use in making recommendations, with rationales

| Articles from evidence summary not included in the guideline: 1. Abercrombie, E. M., Mather, C. A., Hon, J., Graham-King, P., & Pillay, E. (2008). Recessive dystrophic epidermolysis bullosa. Part 2: care of the adult patient. Br J Nurs, 17(6), S6, S8, S10 passim. [5a] No results directly related to interventions for pain, itch, or level of function. 2. Ahmed, A. R., & Dahl, M. V. (2003). Consensus statement on the use of intravenous immunoglobulin therapy in the treatment of autoimmune mucocutaneous blistering diseases. Archives of dermatology, 139(8), 1051–1059. doi: 10.1001/archderm.139.8.1051 [5a] No results directly related to interventions for pain, itch, or level of function. 3. Das, B. B., & Sahoo, S. (2004). Dystrophic epidermolysis bullosa. Journal of Perinatology, 24(1), 41–47. doi: 10.1038/sj.jp.7211019 [5a] No results directly related to interventions for pain, itch, or level of function. 4. Fine, J. D., & Johnson, L. (1988). Evaluation of the efficacy of topical bufexamac in epidermolysis bullosa simplex. A double-blind placebo-controlled crossover trial. Archives of dermatology, 124(11), 1669–1672. Not relevant to current practice 5. Ilić, M. K., Kern, J., Babić, I., Šimić, D., Kljenak, A., & Kogler, V. M. (2011). Application of the procedural consolidation concept to surgical treatment of children with epidermolysis bullosa: A retrospective analysis. Croatian Medical Journal, 52(4), 520–526. doi: 10.3325/cmj.2011.52.520 [4b] No results directly related to interventions for pain, itch, or level of function. 6. Iohom, G., & Lyons, B. (2001). Anaesthesia for children with epidermolysis bullosa: a review of 20 years’ experience. Eur J Anaesthesiol, 18(11), 745–754. [5a] No results directly related to interventions for pain, itch, or level of function. 7. Kirtschig, G., Murrell, D. F., Wojnarowska, F., & Khumalo, N. P. (2009). Interventions for mucous membrane pemphigoid and epidermolysis bullosa acquisita. Cochrane Database of Systematic Reviews(1). [1a] No results directly related to interventions for pain, itch, or level of function. 8. Lara-Corrales, I., Arbuckle, A., Zarinehbaf, S., & Pope, E. (2010). Principles of wound care in patients with epidermolysis bullosa. Pediatr Dermatol, 27(3), 229–237. doi: 10.1111/j.1525-1470.2010.01086.x [5a] No results directly related to interventions for pain, itch, or level of function. 9. Lara-Corrales, I., & Pope, E. (2008). Epidermolysis bullosa: The pediatrician's role. Current Pediatric Reviews, 4(4), 250–257. doi: 10.2174/157339608787407672 [5a] No results directly related to interventions for pain, itch, or level of function. 10. Lin, Y. C., & Golianu, B. (2006). Anesthesia and pain management for pediatric patients with dystrophic epidermolysis bullosa. J Clin Anesth, 18(4), 268–271. doi: 10.1016/j.jclinane.2005.11.004 No results directly related to interventions for pain, itch, or level of function. 11. Meola, S., Olivieri, M., Mirabile, C., & Mastrandrea, P. (2010). Anesthetic management for right upper extremity amputation due to recidivous cutaneous carcinoma and acute postoperative pain control in patients affected by epidermolysis bullosa. Minerva Anestesiol, 76(2), 144–147. [5a] 12. No results directly related to population of interest or interventions for pain, itch, or level of function. 13. Ramamurthi, S., Rahman, M. Q., Dutton, G. N., & Ramaesh, K. (2006). Pathogenesis, clinical features and management of recurrent corneal erosions. Eye (Basingstoke), 20(6), 635–644. doi: 10.1038/sj.eye.6702005 [5a] No results directly related to interventions for pain, itch, or level of function. 14. Sehgal, N., Smith, H., & Manchikanti, L. (2011). Peripherally acting opioids and clinical implications for pain control. Pain Physician, 14(3), 249–258. [5a] No results directly related to interventions for pain, itch, or level of function. 15. Sibbald, R. G., Zuker, R., Coutts, P., Coelho, S., Williamson, D., & Queen, D. (2005). Using a dermal skin substitute in the treatment of chronic wounds secondary to recessive dystrophic epidermolysis bullosa: a case series. Ostomy/wound management., 51(11), 22–46. Not related to the clinical question of interest 16. Sockolov, M. E., Alikhan, A., & Zargari, O. (2009). Non-psoriatic dermatologic uses of monoclonal antibody therapy Monoclonal antibody therapy. Journal of Dermatological Treatment, 20(6), 319–327. doi: 10.3109/09546630902936778 [5a] No results directly related to interventions for pain, itch, or level of function. 17. Stavropoulos, F., & Abramowicz, S. (2008). Management of the Oral Surgery Patient Diagnosed With Epidermolysis Bullosa: Report of 3 Cases and Review of the Literature. Journal of Oral and Maxillofacial Surgery, 66(3), 554–559. doi: 10.1016/j.joms.2007.06.672 [5a] No results directly related to interventions for pain, itch, or level of function. 18. Swartling, C., Karlqvist, M., Hymnelius, K., Weis, J., & Vahlquist, A. (2010). Botulinum toxin in the treatment of sweat-worsened foot problems in patients with epidermolysis bullosa simplex and pachyonychia congenita. British Journal of Dermatology, 163(5), 1072–1076. doi: 10.1111/j.1365-2133.2010.09927.x [4b] Not related to the clinical question of interest 19. Weiner, M. S. (2004). Pain management in epidermolysis bullosa: an intractable problem. Ostomy Wound Manage, 50(8), 13–14. [5b] No results directly related to interventions for pain, itch, or level of function. 20. Wu, J. (2007). Deep sedation with intravenous infusion of combined propofol and ketamine during dressing changes and whirlpool bath in patients with severe epidermolysis bullosa. Paediatr Anaesth, 17(6), 592–596. doi: 10.1111/j.1460-9592.2006.02177.x Not relevant to the population of interest Articles reviewed in full text but not included in the evidence summary or guideline: 1. Bello, Y. M., Falabella, A. F., & Schachner, L. A. (2001). Epidermolysis Bullosa and its Treatment. Wounds, 13(3), 113–118. No results or direction that would inform interventions or outcomes of interest 2. Bhat, A., Naguwa, S. M., Cheema, G. S., & Gershwin, M. E. (2009) Colchicine revisited. Vol. 1173 (pp. 766–773). Not relevant to the clinical question 3. Blanchet-Bardon, C., & Bohbot, S. (2005). Using Urgotul dressing for the management of epidermolysis bullosa skin lesions. Journal of wound care., 14(10), 490–491, 494–496. No results or direction that would inform interventions or outcomes of interest 4. Çagirankaya, L. B., Hatipoglu, M. G., & Hatipoglu, H. (2006). Localized epidermolysis bullosa simplex with generalized enamel hypoplasia in a child. Pediatr Dermatol, 23(2), 167–168. doi: 10.1111/j.1525-1470.2006.00206.x Not relevant to the clinical question 5. Duipmans, J. C., & Jonkman, M. F. (2010). Interdisciplinary management of epidermolysis bullosa in the public setting: The Netherlands as a model of care. Dermatol Clin, 28(2), 383–386. doi: 10.1016/j.det.2010.02.005 Not relevant to the clinical question 6. Fivenson, D. P., Scherschun, L., Choucair, M., KuKuruga, D., Young, J., & Shwayder, T. (2003). Graftskin therapy in epidermolysis bullosa. Journal of the American Academy of Dermatology, 48(6), 886–892. doi: 10.1067/mjd.2003.502 Not relevant to the clinical question 7. Gibson, S., Wilcox, M., Price, P. E., & Clarke, A. J. (2010). Epidermolysis Bullosa exploring the experiences of affected individuals and families of living with an inherited skin condition. Journal of Medical Genetics, 47(Suppl. 1), S103. Not relevant to the clinical question 8. Horn, H. M., & Tidman, M. J. (2000). The clinical spectrum of epidermolysis bullosa simplex. British Journal of Dermatology, 142(3), 468–472. doi: 10.1046/j.1365-2133.2000.03358.x Not relevant to the clinical question 9. Inoue, R., Kobayashi, S., Morimoto, Y., & Kemmotsu, O. (1997). Successful use of propofol for anesthesia for plasty of eyelids in epidermolysis bullosa. Japanese Journal of Anesthesiology, 46(12), 1590–1593. Not relevant to the clinical question 10. Kajbafzadeh, A. M., Mehdizadeh, M., & Shahnavaz, H. (2001). The urological manifestation of junctional epidermolysis bullosa: Possible pathophysiology and a report of successful medical management: A preliminary report. Journal of Urology, 165(5 Supplement), 148. No results or direction that would inform interventions or outcomes of interest 11. Madan, V., & Griffiths, C. E. M. (2007). Systemic ciclosporin and tacrolimus in dermatology. Dermatologic Therapy, 20(4), 239–250. doi: 10.1111/j.1529-8019.2007.00137.x No results or direction that would inform interventions or outcomes of interest 12. Mavili, E., Amaral, J., Healey, A., Karsli, C., Pope, E., & Connolly, B. (2010). Percutaneous interventional radiology procedures in patients with epidermolysis bullosa: Modifications and challenges. American Journal of Roentgenology, 195(2), 468–475. doi: 10.2214/ajr.09.2998 No results or direction that would inform interventions or outcomes of interest 13. McGrath, J. A., Schofield, O. M. V., Ishida-Yamamoto, A., O'Grady, A., Mayou, B. J., Navsaria, H., . . . Eady, R. A. J. (1993). Cultured keratinocyte allografts and wound healing in severe recessive dystrophic epidermolysis bullosa. Journal of the American Academy of Dermatology, 29(3), 407–419. Not relevant to the clinical question 14. Morgan, M., Fine, J., Johnson, L., Stein, A., Suchindran, C., & Weiner, M. (2001). Activities of daily living (ADL) and disease-associated pain in children with inherited epidermolysis bullosa (EB). Journal of Investigative Dermatology, 117(2), 541. No results or direction that would inform interventions or outcomes of interest 15. Mutasim, D. F. (2003). Autoimmune bullous dermatoses in the elderly: Diagnosis and management. Drugs and Aging, 20(9), 663–681. doi: 10.2165/00002512-200320090-00004 Not relevant to the clinical question 16. Naulaers, G., De Jonge, A., Tison, D., Vinckier, F., Casteels, I., Hofman, I., . . . Morren, M. A. (2005). The child with epidermolysis bullosa. Het kind met epidermolysis bullosa, 61(12), 920–928. Not relevant to the clinical question 17. Norup, M. (1999). Treatment of severely diseased newborns: A survey of attitudes among Danish physicians. Acta Paediatrica, International Journal of Paediatrics, 88(4), 438–444. doi: 10.1080/08035259950169846 Not relevant to the clinical question 18. Petek, L. M., Fleckman, P., & Miller, D. G. (2010). Efficient KRT14 Targeting and Functional Characterization of Transplanted Human Keratinocytes for the Treatment of Epidermolysis Bullosa Simplex. Molecular Therapy, 18(9), 1624–1632. doi: 10.1038/mt.2010.102 Not relevant to the clinical question 19. Pillay, E. (2008). Epidermolysis bullosa. Part 1: causes, presentation and complications. Br J Nurs, 17(5), 292–296. No results or direction that would inform interventions or outcomes of interest 20. Scheinfeld, N. (2003). Phenytoin in cutaneous medicine: Its uses, mechanisms and side effects. Dermatology Online Journal, 9(3), 83–106. Not relevant to the clinical question 21. Schiavo, A. L., Puca, R. V., Ruocco, V., & Ruocco, E. (2010). Adjuvant drugs in autoimmune bullous diseases, efficacy versus safety: Facts and controversies. Clinics in Dermatology, 28(3), 337–343. doi: 10.1016/j.clindermatol.2009.06.018 Not relevant to the clinical question 22. Sezgin, G., Ceyhan, M., Secmeer, G., Bakkaloglu, A., Kanra, G., & Buyukkale, G. (1999). Leukocytoclastic vasculitis in a child with epidermolysis bullosa simplex. Turkish Journal of Pediatrics, 41(2), 277–282. Not relevant to the clinical question 23. Siañez-González, C., Pezoa-Jares, R., & Salas-Alanis, J. C. (2009). Congenital epidermolysis bullosa: A review. Epidermólisis ampollosa congénita: Revisión del tema, 100(10), 842–856. doi: 10.1016/s0001-7310(09)72912-6 Not relevant to the clinical question 24. Takano, H., Kawase, M., Takahashi, T., & Shimada, Y. (2000). Spinal anesthesia for a patient with epidermolysis bullosa hereditaria dystrophica. Japanese Journal of Anesthesiology, 49(9), 1018–1020. Not relevant to the clinical question 25. Venugopal, S. S., Intong, L. R., Cohn, H. I., Mather-Hillon, J., & Murrell, D. F. (2010). Responsiveness of nonHerlitz Junctional Epidermolysis Bullosa to topical gentian violet. Int J Dermatol, 49(11), 1282–1285. doi: 10.1111/j.1365-4632.2010.04557.x Not relevant to the clinical question Yan, E. G., Paris, J. J., Ahluwalia, J., Lane, A. T., & Bruckner, A. L. (2007). Treatment decision-making for patients with the Herlitz subtype of junctional epidermolysis bullosa. Journal of Perinatology, 27(5), 307–311. doi: 10.1038/sj.jp.7211694 No results or direction that would inform interventions or outcomes of interest |
| --- |
