# Supplementary figures and images for: Pain care for patients with epidermolysis bullosa: best care practice guidelines
Source: BMC Med. 2014 Oct 9;12:178. doi: 10.1186/s12916-014-0178-2 (PMC4190576; doi:10.1186/s12916-014-0178-2)

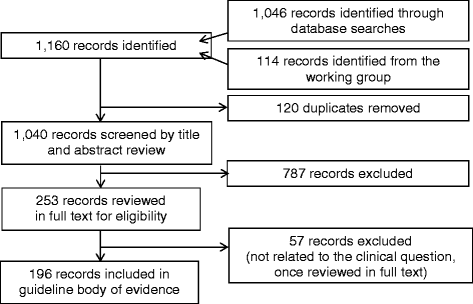

Supplement: Supplementary file 2 — Authors’ original file for figure 1 [file 12916_2014_178_MOESM2_ESM.gif]
